# Epilepsy miRNA Profile Depends on the Age of Onset in Humans and Rats

**DOI:** 10.3389/fnins.2020.00924

**Published:** 2020-09-15

**Authors:** Jiri Baloun, Petra Bencurova, Tereza Totkova, Hana Kubova, Marketa Hermanova, Michal Hendrych, Martin Pail, Sarka Pospisilova, Milan Brazdil

**Affiliations:** ^1^Central European Institute of Technology, Masaryk University, Brno, Czechia; ^2^Brno Epilepsy Center, Department of Neurology, Medical Faculty of Masaryk University, St. Anne’s University Hospital, Brno, Czechia; ^3^Department of Developmental Epileptology, Institute of Physiology of the Czech Academy of Sciences, Prague, Czechia; ^4^First Department of Pathology, Medical Faculty of Masaryk University, St. Anne’s University Hospital, Brno, Czechia

**Keywords:** miRNA, mesial temporal lobe epilepsy, animal model, human, sequencing, cross-comparison study

## Abstract

Temporal lobe epilepsy (TLE) is a severe neurological disorder accompanied by recurrent spontaneous seizures. Although the knowledge of TLE onset is still incomplete, TLE pathogenesis most likely involves the aberrant expression of microRNAs (miRNAs). miRNAs play an essential role in organism homeostasis and are widely studied in TLE as potential therapeutics and biomarkers. However, many discrepancies in discovered miRNAs occur among TLE studies due to model-specific miRNA expression, different onset ages of epilepsy among patients, or technology-related bias. We employed a massive parallel sequencing approach to analyze brain tissues from 16 adult mesial TLE (mTLE)/hippocampal sclerosis (HS) patients, 8 controls and 20 rats with TLE-like syndrome, and 20 controls using the same workflow and categorized these subjects based on the age of epilepsy onset. All categories were compared to discover overlapping miRNAs with an aberrant expression, which could be involved in TLE. Our cross-comparative analyses showed distinct miRNA profiles across the age of epilepsy onset and found that the miRNA profile in rats with adult-onset TLE shows the closest resemblance to the profile in mTLE/HS patients. Additionally, this analysis revealed overlapping miRNAs between patients and the rat model, which should participate in epileptogenesis and ictogenesis. Among the overlapping miRNAs stand out miR-142-5p and miR-142-3p, which regulate immunomodulatory agents with pro-convulsive effects and suppress neuronal growth. Our cross-comparison study enhanced the insight into the effect of the age of epilepsy onset on miRNA expression and deepened the knowledge of epileptogenesis. We employed the same methodological workflow in both patients and the rat model, thus improving the reliability and accuracy of our results.

## Introduction

Temporal lobe epilepsy (TLE) is the most common type of epilepsy—a neurological disorder characterized by recurrent spontaneous seizures. This condition includes several subtypes, of which the most prevalent is mesial TLE (mTLE). The seizures of this subtype originate from the structures in the medial portion of the temporal lobe (such as the hippocampus) and are often associated with a pathological change of the hippocampal tissue—hippocampal sclerosis (HS) (Thom et al., 2009). The underlying mechanisms of epileptogenesis in TLE are still unclear; hence, a better understanding of involved biological processes is fundamental for novel treatment strategies and therapeutics. Among potential therapeutic targets as well as possible biomarkers of pathological changes in the epileptic brain are microRNAs (miRNAs).

miRNAs are small endogenous noncoding RNAs that act as posttranscriptional regulators of gene expression. Individual miRNAs can target a variety of mRNAs and most commonly inhibit their translation into proteins (Bartel, 2009). Hence, various mechanisms (e.g., methylation hormones and other miRNAs) strictly regulate miRNA expression (review in Gulyaeva and Kushlinskiy, 2016), because their dysregulation can have a profound effect on the cell, and, subsequently, on the whole organism. As a result, the monitoring of miRNA expression might disclose the onset or progression of the disease, making miRNAs a useful biomarker. This relation illustrates the close link between many diseases and altered levels of miRNAs (Srinivasan et al., 2013). The changes in miRNome have been mostly studied in different types of cancer (Li and Kowdley, 2012), but the connection occurs in other conditions, including TLE. Numerous studies of miRNA dysregulation in TLE have provided solid evidence for their importance in the epilepsy pathogenesis (Brennan and Henshall, 2018).

To date, many studies about the role of miRNAs in epilepsy have produced a vast amount of data, however, they also have several drawbacks. Most of them studied epilepsy only in animal models, which have important limitations. Firstly, the animal model is only an approximation of mTLE, which does not correspond perfectly to the biochemistry of this disease in humans. Therefore, the results require cautious interpretation in the context of human mTLE. Moreover, there are fundamental differences between the animal models as each of them implements a different technique to induce epileptic seizures, making it difficult to compare the acquired data (Korotkov et al., 2017). Different experimental protocols can lead to the activation of different pathological pathways. Therefore, the processes involved in epileptogenesis can vary from one model to another. As a result, the affected miRNAs can also be unique to the experimental model and not represent epilepsy in general. Indeed, the results of these studies are often discordant (Korotkov et al., 2017).

Another fraction of the studies focuses on analyzing the miRNome of human brain samples from epilepsy surgeries. This approach has the potential to provide results fully relevant to human mTLE, however, it is not flawless. Unlike TLE patients, controls were not treated with antiepileptic drugs (AEDs) affecting miRNA expression in the brain tissue. Furthermore, some complex factors can never be fully accounted for (such as lifestyle, age, gender, or ethnicity) (Leung and Sharp, 2010; Dluzen et al., 2016; Huan et al., 2018). These factors might affect the miRNA composition of the hippocampal tissue (Crespo et al., 2018) and cause inter-individual variability in both control and patient groups. Another discussed drawback is the origin of control brain tissues, which mostly come from postmortem autopsies, however, previous research discovered virtually unchanged miRNA composition in postmortem tissues within 30 h after death (Kakimoto et al., 2015; Bencurova et al., 2017).

The most promising approach is to compare the data acquired in animal models and human patients and find the intersection. Additionally, the same methodological workflow could enhance reliability and reduce the false negatives in the results. miRNAs identified by this approach are most likely related to epilepsy, rather than to a specific model or the origin of the tissue (surgery or autopsy). To date, only a few studies have used this approach (Roncon et al., 2015; Korotkov et al., 2017); therefore, we compared the miRNomes of patients with mTLE and a rat model of chronic epilepsy from our previous experiments (Bencurova et al., 2017). Moreover, the miRNA expression profile in the brain tissues changes during the development, and seizures might affect the direction of these changes. Our analyses also evaluated the effect of age at the first seizure occurrence on the miRNA profile in the chronic phase of this disease.

## Materials and Methods

### Human Samples

All procedures involving human tissue processing were approved by the Ethical Committee of St. Anne’s University Hospital in Brno (approval number 9G/2015-KS). The collection of the hippocampal tissue was performed on a cohort of anteromesial temporal resections at St. Anne’s University Hospital in Brno, the Czech Republic, within the period 2007–2016. Patients and controls were previously described in Bencurova et al. (2017). In this study, we focused on a cohort of 16 adult mTLE/HS patients and 8 controls, hippocampal tissues of which were utilized for whole-miRNome analysis by massive parallel sequencing (MPS). All patients (seven men and nine women) were referred to the Department of Neurology at the Brno Epilepsy Center for their medical intractability and fulfilled the diagnostic criteria for mTLE/HS. The diagnosis was made according to the International League Against Epilepsy (ILAE) criteria (Commission on Classification and Terminology of the ILAE, 1989). All of the patients had been routinely investigated, including long-term semi-invasive video-EEG monitoring (using sphenoidal electrodes), high-resolution magnetic resonance imaging (MRI), and neuropsychological testing. The diagnosis of unilateral mTLE in our patients was based on a consonance of history data, ictal and interictal EEG findings, ictal semiology, neuropsychology, and neuroimaging findings (MRI and PET). Unilateral HS concordant with the electroencephalographic lateralization of the epileptogenic zone was confirmed in all cases by visual inspections of the MRI scans by two independent physicians. None of the patients revealed other brain structural lesions on MRI scans or had undergone previous intracranial surgery. Age of TLE onset ranged from 2 to 44 years (mean = 16.9), and the average patient’s age at the time of surgery was 40.2 years (range = 25–51). All patients signed informed consent to approve the use of their tissue in this study. Control hippocampal tissue was collected from eight postmortem cases without hippocampal aberrations from the Department of Forensic Medicine at St. Anne’s University Hospital. The average age of these cases at the time of death was 55.6 years (range = 30–72). All procedures to obtain hippocampal control tissue samples were in accordance with the Czech Republic’s legislation and ethical standards and with the 1964 Helsinki declaration (revised in 2013). A detailed overview of patients and controls is listed in Supplementary Table S1.

### The Rat Model of TLE

Protocols involving animals were performed in accordance with ARRIVE guidelines (Kilkenny et al., 2010) and national (Act No 246/1992 Coll.) and international laws and policies (EU Directive 2010/63/EU for animal experiments). The Ethical Committee of the Czech Academy of Sciences approved the experimental protocols (Approval No. 128/2013). Status epilepticus (SE) was induced in male Wistar albino rats aged P12 (P—postnatal day) or P60 as described previously by Kubová et al. (2004). Briefly, all animals were labeled with a unique code and injected intraperitoneally (i.p.) with 127 mg/kg LiCl 24 h prior to pilocarpine/saline injection. Individual animals were tracked throughout the entire project based on the assigned code of each animal. Control and SE groups contained randomly assigned animals, and subjects in the SE group received a single i.p. dose of pilocarpine at P12 (35 mg/ml/kg) or P60 (45 mg/ml/kg); controls were treated with saline. All treated animals were caged separately and continually observed for 3 h. Latency to motor SE characterized by forelimb clonus was registered. Animals were treated with paraldehyde (P12: 0.07 ml/kg and P60: 0.3 ml/kg) approximately 2 h after the onset of convulsive SE in order to decrease mortality. Further experiments included only rats exhibiting behavioral manifestations of seizures progressing to forelimbs clonus for at least 1 h (SE was successfully induced in 100% of P12 and 69% of P60 animals injected with pilocarpine). Twelve P12 and 13 P60 animals were selected for experiments in the chronic stage of epilepsy (3 months after SE), along with 10 control animals per age group. For more details, see Supplementary Methods (submitted manuscript).

### Monitoring

One week before being sacrificed, animals underwent continuous 24/7 video monitoring with IP infrared Camera Edimax IC-3140W for wireless monitoring. Synology Surveillance Station 7 software was used for both registration and evaluation. An experienced observer evaluated the recordings manually, registering the incidence of motor seizures (Racine stages 3–5). The electrographic analysis was omitted in order to prevent possible inflammatory reactions on EEG electrode implantation and the adverse effect of anesthesia exposure. The remainder of adult-onset animals (*n* = 10) exhibited similar clonic seizures (frequency summarized in Supplementary Figure S1), while animals with SE at P12 and controls did not exhibit motor seizures (submitted manuscript).

### Hippocampal Tissue Processing and RNA Isolation

Surgically resected and autopsy tissues from adult mTLE/HS patients and controls were identically treated: fixed in 10% neutral buffered formalin, grossly inspected, carefully oriented, and measured. Hippocampal tissue specimens were dissected into 2–3 mm-thick tissue slices along the anterior–posterior axis and paraffin embedded. Formalin-fixed paraffin-embedded (FFPE) tissue sections were stained with hematoxylin–eosin and evaluated under light microscopy; additionally, the presence of neuronal depletion and gliosis in HS tissue samples was confirmed using NeuN and GFAP immunohistochemistry. The international consensus classification of HS in TLE was applied (Blümcke et al., 2013). For miRNA analysis, paraffin-embedded tissue slices showing the presence of hippocampal complex were selected. Total RNA was isolated from tissue sections using the High Pure miRNA Isolation Kit (Roche) according to the manufacturer’s protocol. In order to maximize RNA yield, overnight Proteinase K digestion was used.

Hippocampal tissue was collected from 10 SE and 10 control animals per age group (*n* = 40) 3 months after SE (chronic stage of epilepsy). Animals were sacrificed by decapitation under the overdose of anesthesia (ether). Brains were immediately dissected, and the entire hippocampus was collected from both hemispheres. Tissues were immediately frozen in dry ice and stored at −80°C until further processing. Total RNA was isolated from frozen hippocampal tissue using the TRI Reagent^®^ (Biotech) according to the manufacturer’s protocol. Ceramic beads were used for tissue disruption.

RNA was successfully extracted from all specimens and quantified using NanoDrop ND-1000 (Thermo Fisher Scientific) and Qubit^TM^ dsDNA HS Assay Kit on a Qubit^®^ 2.0 fluorometer (Thermo Fisher Scientific). The 1.1 μg of extracted total RNA was used to prepare small RNA libraries and sequenced on NextSeq 500 (Illumina).

### Massive Parallel Sequencing

Libraries for sequencing were prepared from 1.1 μg of total RNA obtained from rat samples (20 SE and 20 controls) and patients (16 mTLE and 8 controls). Libraries were prepared using a NEXTflex Small RNA-Seq Kit v2 for human samples and Kit v3 for rat samples (Bioo Scientific) according to the manufacturer’s protocol. After the amplification step, samples were analyzed using a Fragment Analyzer (Advanced Analytical), and specific fragments of the miRNA library (145 bp) were quantified. Samples were equally pooled for each particular sequencing run based on the concentration of miRNA library fragments, which were isolated using the Pippin Prep instrument using the 3% agarose (Sage Science) prior to sequencing. Isolated fragments were quantified using NanoDrop ND-1000 (Thermo Fisher Scientific) and Qubit^®^ 2.0 fluorometer (Thermo Fisher Scientific) and used for sequencing on NextSeq 500 (Illumina) according to the manufacturer’s protocol.

### MPS Data Processing and Differential Expression Analysis

Human sequencing data were processed as described previously (Bencurova et al., 2017). Adapter trimming, quality control, and miRNA annotation against miRBase v21 were performed using the *Chimira* (Vitsios and Enright, 2015) tool. Further analyses were performed using *R/Bioconductor* packages (R Core Team, 2020).

MPS data from the rat TLE model were analyzed individually for each age group. Adaptor sequences were scanned and identified by the Kraken package (v15-065) (Davis et al., 2013) and removed with Cutadapt (v1.12) software (Martin, 2011). Only high-quality reads (Phred ≥ 10 over at least 85% of the read length) with a length between 16 and 28 bp after adapter trimming were retained as potential miRNA reads. The quality of both raw and processed reads was evaluated using FastQC software (Andrews, 1973). The raw miRNA expression levels were quantified by seqBuster (1.2.4a6) (maximum of one mismatch) with miRBase annotation (v22) (Kozomara and Griffiths-Jones, 2014).

Differential expression analysis was evaluated for both human and rat data by R package DESeq2 (Love et al., 2014). Raw data and annotated sequences of the small RNA libraries can be found in the GEO database (accession numbers GSE99455 and GSE124332).

The expression of miRNA was determined by microRNA quantitative PCR (miQPCR) as described by Benes et al. (2015). Primers were either designed manually or downloaded from the list of miQPCR primers, validated, and optimized for our samples according to the MIQE Guidelines (Bustin et al., 2009). The protocol used for amplification, quantification, and evaluation of miRNA expression using miQPCR was previously described in Bencurova et al. (2017).

### miRNA Target Prediction

Target prediction tools were applied to address putative mRNA targets (miRDB - MicroRNA Target Prediction And Functional Study Database) (Wong and Wang, 2015) and pathways (DIANA-mirPath v3) affected by miRNAs with altered regulation in both mTLE/HS patients and the rat model of TLE. Only miRNAs showing the same trend of dysregulation (upregulated/downregulated) in both species were included in target and pathway prediction. Pathway prediction was based on predicted mRNA targets and experimentally validated miRNA interactions from *DIANA-TarBase* (Vlachos et al., 2015). Both MirTarget and DIANA-mirPath analyzed specific miRNA targets for species *Rattus norvegicus* and *Homo sapiens sapiens* individually.

## Results

### Whole miRNome Sequencing

Our first high-throughput screening focused on profiling the miRNA expression in mTLE/HS patients and controls in order to identify aberrantly expressed miRNAs in this disease (Bencurova et al., 2017). Briefly, we sequenced the hippocampi of 16 patients and eight postmortem controls with median raw reads of 10.8 million per sample. The number of reads after adapter trimming and miRNA mapping dropped to 1.5 million per sample (12% of total) with high quality of sequencing represented by the PHRED-like score above 30 for 98.5% reads. The MPS analyses detected 401 miRNAs with the coverage over 500 reads in all samples together. The total sum of ambiguous base content was below 0.01% of all bases. Prior to the interpretation of differential expression analysis, we conducted additional analysis of gender- and age-related miRNAs in our dataset in order to prevent bias arising from uneven distribution of age and gender in patient and control groups. These analyses did not uncover any gender-specific miRNA with an altered expression level in mTLE/HS within our data, while miR-7110-3p upregulated (*p* = 0.028) in controls > 60 years of age was removed from follow-up analyses (data not shown). In our previous work, we described that miRNA distribution is impartial to the autopsy delay from the death of the subject and that the cellular composition of hippocampi is similar (based on the analysis of miRNAs enriched in specific cell types) in our patients and controls (Bencurova et al., 2017).

Parallel to human MPS, we sequenced rat hippocampi in the chronic stage of epileptogenesis (submitted manuscript). The final median of clean potential miRNA reads was 2.6 million (∼53.91% of the raw reads) after adapter trimming, size selection of the trimmed reads, and removal of possible contaminants. Mapping to *miRBase* (v22) revealed the median content of miRNAs in the samples of 2.3 million (50.45% of the raw reads). Sequencing identified 412 unique miRNA species with read coverage > 500 across all the samples. The average PHRED-like quality score of all samples was above 35, which indicated very high sequencing quality. The total sum of ambiguous base content was < 0.01%. Since all rat individuals were age-matched males, housed under the same conditions, additional correlation analyses were unnecessary.

### miRNA Expression in TLE Patients and Model

**FIGURE 1:**
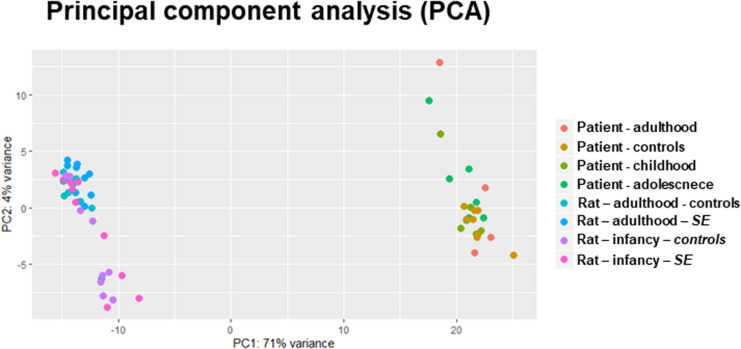
PCA of whole-miRNome expression in rats and humans. The PCA is a transformation technique to describe the variation of miRNA expression (normalized read counts) and thus can detect the correlation and clustering in data. Our PCA plot indicates distinctive miRNA expression between patients and rat models of epilepsy.

Principal component analysis (PCA) visualizes variation and uncovers distinct patterns in a dataset. This analysis showed different miRNA production between patients and rats – human individuals clustered separately from rats without any apparent effect of the age of TLE onset on the overall miRNA expression (Figure 1). Indeed, analysis of the differential expression using DESeq2 showed that only a fraction of over 400 identified miRNAs reached the threshold of differential expression (*p* < 0.05, fold change of upregulation or downregulation exceeding 1.4 and a minimum of 500 reads) in both human (Supplementary Table S2) and rat TLEs (Supplementary Table S3).

The basic concept suggests that the presence of seizures in childhood and adolescence could affect the miRNA expression profile and thus potentially introduce a defect in brain development (reviewed in Henshall, 2014; Rao and Pak, 2016). For this reason, we categorized and analyzed patient data in four categories based on their epilepsy onset age: (a) all patients together; (b) the first unprovoked seizure before the age of 10 (childhood); (c) between the 10th and 19th years (adolescent); and (d) at the age of 20 or later (adult onset).

**FIGURE 2:**
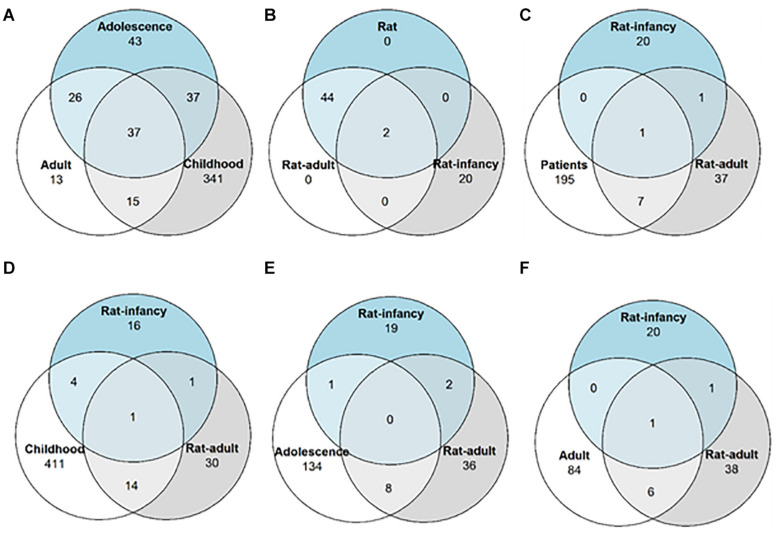
Differentially expressed miRNA distribution across TLE onset stages. Venn diagrams showing the number of overlapping miRNAs with altered expression among **(A)** patients with different epilepsy onset ages; **(B)** both the rat model with different onset age and the rat model without onset age categorization; **(C)** patients without categorization and two rat models with different onset ages; **(D)** patients with epilepsy onset in infancy and both rat models of onset age; **(E)** patients with epilepsy onset in adolescence and both rat models of onset age; and **(F)** patients with epilepsy onset in adulthood and both rat models of onset age.

The differential expression analysis of patients without respect to the first seizure was described in our previous study (Bencurova et al., 2017) and was focused on the detection of dysregulated miRNAs in mTLE/HS patients whose age of the first unprovoked seizure was from 2 to 44 years. This group included mainly miRNAs previously associated with epilepsy, while nine of them have been described in epilepsy for the first time (Bencurova et al., 2017). Next, we searched in our MPS datasets for differentially expressed miRNAs in patients with the TLE onset between the ages of 2 and 9 years and discovered 123 miRNAs significantly dysregulated in patients. In the adolescence-onset category, 130 miRNAs were significantly dysregulated in TLE. The category with onset age above 20 contained 80 dysregulated miRNAs (Supplementary Table S2). Of note, 49 miRNAs were significantly dysregulated in all age categories (Figure 2).

Since all patients underwent surgery in adulthood, the period between epilepsy onset and sample collection differed among childhood-onset (mean = 33.2 years, *SD* = 10.3), adolescent-onset (mean = 23 years, *SD* = 9.3), and adult-onset (mean = 11 years, *SD* = 11.9) categories. The duration of epilepsy might have affected the expression of detected miRNA, and correlations were found between the expression of miR-142-3p, miR-135a-5p, and miR-484 with the duration of epilepsy, but their effects were low, as displayed in Supplementary Figures S2, S4.

DESeq2 analysis of all rat samples combined identified 19 miRNAs differentially expressed in the post-SE rat (*p* < 0.05, minimal fold change of 1.4 with read count over 500 across all samples). When categorized by the onset age, 42 miRNAs showed altered expression in adult-onset and 12 in the infancy-onset model of TLE (Figure 2). However, only rno-miR-24-2-5p and miR-135a-5p were upregulated in both age groups (Supplementary Table S3; submitted manuscript).

To identify dysregulated miRNAs shared between patients and our animal model, we compared categorized rat miRNA profiles (infancy and adult onset) with data in each onset age category of patients. This comparison showed 19 miRNAs with significantly altered expression in both patients and TLE rats (Table 1), but seven of these miRNAs showed the opposite direction of regulation in rats compared with patients (Table 2). The overlap between human miRNA profile and the combined ages of onset categories in the rat was minimal. miR-142-3p showed common dysregulation across all patient categories and rat adult-onset group, and miR-135a-5p was dysregulated in rats (both age groups) and patients with epilepsy onset in adulthood (Table 1). miR-142-5p was dysregulated in patients with epilepsy onset in infancy, adolescence, and patients without categorization (all onset ages). Altogether, rats with SE in adulthood showed higher similarity with mTLE/HS patients in all categories, while the infancy-onset group corresponded with the human profile only in the case of miR-135a-5p and miR-140-5p (Figure 2).

**TABLE 1:** miRNA commonly dysregulated in mTLE/HS patients and TLE-like rats.

		**MPS**		**miQPCR**	
**miRNA**	**Age category**	***p*-value**	**Fold change**	***p*-value**	**Fold change**
**(A)**					
let-7b-3p	All onset ages	**0.047**	**1.73**	0.070	1.28
	2–9 years (childhood)	0.105	1.68	0.275	1.25
	10–19 years (adolescence)	**0.027**	**2.53**	**0.042**	**1.46**
	≥ 20 years (adult)	0.266	**2.32**	0.794	1.06
miR-129-2-3p	All onset ages	**0.000**	**3.19**	**0.006**	**2.12**
	2–9 years (childhood)	**0.000**	**4.11**	**0.002**	**2.60**
	10–19 years (adolescence)	**0.000**	**4.12**	**0.008**	**2.18**
	≥ 20 years (adult)	**0.000**	**3.10**	**0.034**	**2.34**
miR-130b-3p	All onset ages	**0.005**	**−1..07**	0.053	**1.49**
	2–9 years (childhood)	0.062	**−1..38**	0.166	**1.52**
	10–19 years (adolescence)	0.063	**−1..33**	0.076	**1.62**
	≥ 20 years (adult)	**0.000**	**−1..45**	0.566	1.24
miR-135a-5p	All onset ages	**0.000**	**1.61**	0.114	**1.50**
	2–9 years (childhood)	0.151	**1.42**	0.687	1.13
	10–19 years (adolescence)	0.099	**1.54**	**0.046**	**1.73**
	≥20 years (adult)	**0.026**	**1.75**	0.209	**1.69**
miR-140-5p	All onset ages	0.105	1.05	0.180	1.27
	2–9 years (childhood)	**0.012**	1.38	0.665	1.10
	10–19 years (adolescence)	**0.013**	**1.41**	**0.013**	**1.57**
	≥20 years (adult)	0.889	–1.27	0.872	1.04
miR-142-3p	All onset ages	**0.000**	**3.22**	**0.003**	**2.53**
	2–9 years (childhood)	**0.000**	**2.09**	**0.010**	**2.46**
	10–19 years (adolescence)	**0.000**	**2.42**	**0.010**	**2.46**
	≥20 years (adult)	**0.000**	**2.19**	**0.035**	**2.14**
miR-142-5p	All onset ages	**0.000**	**2.66**	**0.004**	**2.34**
	2–9 years (childhood)	**0.001**	**2.10**	**0.010**	**2.68**
	10–19 years (adolescence)	**0.006**	**1.87**	**0.009**	**2.77**
	≥ 20 years (adult)	**0.002**	**1.90**	**−**	**−**
miR-193a-5p	All onset ages	**0.011**	**1.63**	**0.000**	**2.46**
	2–9 years (childhood)	**0.004**	**1.84**	0.137	**1.56**
	10–19 years (adolescence)	**0.010**	**1.83**	**0.036**	**2.21**
	≥ 20 years (adult)	0.098	**1.59**	0.456	–1.21
miR-203a-3p	All onset ages	**0.003**	**1.79**	**0.000**	**2.20**
	≥ 20 years (adult)	0.302	1.14	0.068	**1.78**
	2–9 years (childhood)	**0.000**	**1.99**	**0.032**	**1.95**
	10–19 years (adolescence)	**0.002**	**1.97**	**0.001**	**2.62**
miR-484	All onset ages	0.013	1.05	0.025	**2.54**
	2–9 years (childhood)	0.004	1.34	0.151	**2.79**
	10–19 years (adolescence)	0.008	**1.40**	0.214	**2.16**
	≥ 20 years (adult)	0.002	**1.46**	0.169	**3.02**
miR-490-5p	All onset ages	0.041	1.07	0.916	1.03
	2–9 years (childhood)	0.103	1.13	0.987	1.01
	10–19 years (adolescence)	0.021	1.38	0.450	1.28
	≥ 20 years (adult)	0.002	**1.44**	0.290	–1.39
miR-539-5p	All onset ages	0.025	**−1..53**	0.182	**2.25**
	2–9 years (childhood)	0.001	**−1..73**	0.378	**2.64**
	10–19 years (adolescence)	0.000	**−1..86**	0.431	**2.07**
	≥ 20 years (adult)	0.433	–1.19	0.537	**2.13**
**(B)**					
let-7b-3p	All onset ages	0.133	1.39	0.920	**−1..01**
	P12 (infant)	0.948	**−1..01**	0.811	**−1..05**
	P60 (adult)	**0.001**	**1.58**	0.886	1.02
miR-129-2-3p	All onset ages	**0.025**	1.23	0.102	1.13
	P12 (infant)	0.657	1.09	0.177	1.12
	P60 (adult)	**0.005**	**1.66**	0.182	1.15
miR-130b-3p	all onset ages	**0.001**	**−2.07**	0.770	**−1..05**
	P12 (infant)	0.571	**−1..25**	0.664	1.12
	P60 (adult)	**0.002**	**−3.48**	0.120	**−1..24**
miR-135a-5p	All onset ages	**0.000**	**1.50**	0.093	1.23
	P12 (infant)	**0.007**	**1.46**	0.211	**1.49**
	P60 (adult)	**0.001**	**1.58**	0.314	1.16
miR-140-5p	All onset ages	**0.014**	**1.40**	0.722	**−1..02**
	P12 (infant)	**0.018**	**1.42**	0.224	1.11
	P60 (adult)	0.099	1.27	0.100	**−1..17**
miR-142-3p	All onset ages	**0.040**	1.23	0.716	**−1..03**
	P12 (infant)	0.312	1.24	0.742	1.04
	P60 (adult)	**0.040**	**1.54**	0.332	**−1..09**
miR-142-5p	All onset ages	0.075	1.36	**−**	–
	P12 (infant)	0.989	**−1..00**	0.109	**1.56**
	P60 (adult)	**0.012**	**1.58**	**0.010**	**2.81**
miR-193a-5p	All onset ages	**0.008**	1.24	0.753	**−1..07**
	P12 (infant)	0.152	1.33	**0.000**	**−1..65**
	P60 (adult)	**0.026**	**1.58**	0.145	**1.45**
miR-203a-3p	All onset ages	**0.018**	1.24	0.904	1.01
	P12 (infant)	0.711	**−1..05**	0.630	**−1..09**
	P60 (adult)	**0.000**	**1.57**	0.368	1.11
miR-484	All onset ages	0.057	**1.51**	0.121	1.23
	P12 (infant)	0.754	1.08	0.579	**1.51**
	P60 (adult)	**0.009**	**1.87**	**0.023**	**1.53**
miR-490-5p	All onset ages	0.460	1.09	0.113	**−1..23**
	P12 (infant)	0.471	**−1..27**	**0.001**	**−1..58**
	P60 (adult)	**0.039**	**1.99**	0.733	1.04
miR-539-5p	All onset ages	0.081	**−1..09**	**0.017**	**−1..22**
	P12 (infant)	0.960	1.01	0.563	**−1..06**
	P60 (adult)	**0.028**	**−1..43**	**0.000**	**−1..41**

*List of miRNAs with altered expression determined by MPS and their miQPCR validation in **(A)** mTLE/HS patients and **(B)** rats 3 months after SE. miRNAs are displayed for each analyzed onset age category and all samples per species together (altogether 12 unique miRNAs). p-values below 0.05 are in bold. Fold changes < −1.4 or > 1.4 are in bold. Empty cells indicate impossibility to analyze miRNA expression due to high Ct values (>32) in miQPCR.*

**TABLE 2:** miRNA with opposite dysregulation in mTLE/HS patients and TLE-like rats.

	**Human mTLE + HS**				**Rat 3 months after SE**		
**First seizure**	**miRNA**	***p*-value**	**FC**	**Age at SE**	**miRNA**	***p*-value**	**FC**
All onset ages	miR-22-3p	**	–1.52	All onset ages	miR-187-3p	**	–1.48
	miR-301a-3p	**	2.22		miR-211-5p	**	1.83
2–9 years (childhood)	miR-22-3p	**	–1.81		miR-301a-3p	**	–1.33
	miR-301a-3p	**	1.79		miR-376a-3p	*	–1.34
10–19 years (adolescence)	miR-187-3p	*	1.73		miR-92b-5p	*	–1.37
	miR-211-5p	**	–3.29	P12 (infant)	miR-22-3p	*	1.89
	miR-212-3p	**	–2.22		miR-301a-3p	*	–1.46
	miR-22-3p	**	–1.83	P60 (adult)	miR-187-3p	**	–2.05
	miR-301a-3p	**	1.91		miR-212-3p	**	1.69
*≥20 years (adult)*	miR-301a-3p	**	2.26				
	miR-211-5p	**	–4.96				
	miR-376a-3p	**	1.97				
	miR-92b-5p	*	2.00				

List of miRNAs with altered expression (**p-value < 0.01; *p-value < 0.05; fold change over 1.4) with an opposite trend of dysregulation in mTLE/HS patients and rats 3 months after SE screened by MPS. miRNAs are displayed for each analyzed onset age category and all samples per species together (altogether seven unique miRNAs). FC—differential expression fold change.

In patient samples, miQPCR validated the dysregulation of miR-129-2-3p and miR-142-3p across all onset categories in patients, and let-7b-3p, miR-135-5p, and miR-140-5p increased in patients with TLE onset in adolescence (Table 1). Further, miQPCR confirmed increased expressions of miR-142-5p, miR-193-5p, and miR-203a-3p in one or more onset groups in patients along with the group without onset categorization. In the rat samples, miQPCR validated three out of 12 miRNAs, which overlapped with patients in their altered expression in TLE in our sequencing results: miR-142-5p, miR-484, and miR-539-5p in the adult-onset group.

### Putative Targets of Dysregulated miRNAs

The MirTarget search tool (miRDB-MicroRNA Target Prediction And Functional Study Database) produced a list of putative mRNA targets of all identified miRNAs with shared dysregulation in SE animals and mTLE + HS patients. Table 3 shows brain-function-related targets with a target score of 90 or higher for humans and rats. Among the genes targeted in humans, the most frequent are potassium channels regulated by five miRNAs (miR-130b-3p, miR-135a-5p, miR-129-2-3p, miR-539-5p, and miR-484), followed by semaphorins and solute carriers of various molecules targeted by let-7b-3p and miR-135a-5p, miR-142-5p, and miR-539-5p. Solute carriers stand out also among mRNA targets in rats with the same number of miRNAs involved in their regulation (let-7b-3p and miR-135a-5p, miR-142-5p, and miR-203a-3p). Unlike those in humans, calcium and sodium channels occur more frequently among targets than potassium channels, which are regulated only by miR-135a-5p in rats.

**TABLE 3:** Predicted targets of miRNA commonly dysregulated in human and rat TLEs.

**miRNA**	**Human**		**Rat**	
	**Gene**	**Product**	**Gene**	**Product**
let-7b-3p	BDNF	Brain-derived neurotrophic factor	BDNF	Brain-derived neurotrophic factor
	CACNA1C	Calcium voltage-gated channel subunit alpha 1 C	GLS	Glutaminase
	CAMTA1	Calmodulin binding transcription activator 1	NPY1R	Neuropeptide Y receptor Y1
	CARF	calcium responsive transcription factor	SCN7a	Sodium voltage-gated channel alpha subunit 7
	CEPT1	Choline/ethanolamine phosphotransferase 1	SLC18A2	Solute carrier family 18 member A2 (monoamines)
	GABRG1	Gamma-aminobutyric acid type A receptor gamma 1 subunit	SYNGR3	Synaptogyrin 3 (synaptic vesicles)
	GLS	Glutaminase		
	GPR37	G protein-coupled receptor 37		
	GRM5	Glutamate metabotropic receptor 5		
	MNX1	Motor neuron and pancreas homeobox 1		
	NBEA	Neurobeachin (neuron specific post-Golgi membrane traffic)		
	NEUROG2	Neurogenin 2 (differentiation and survival of dopaminergic neurons)		
	NGDN	Neuroguidin (development of nervous system)		
	NLGN1	Neuroligin 1 (formation and remodeling of synapses)		
	NPY	Neuropeptide Y		
	NRP2	Neuropilin 2 (axon guidance)		
	PSD2	Pleckstrin and Sec7 domain containing 2 (endocytosis)		
	SCAMP1	Secretory carrier membrane protein 1		
	SEMA3C	semaphorin 3C		
	SLC18A2	Solute carrier family 18 member A2 (monoamines)		
	SLC6A17	Solute carrier family 6 member 17 (presynaptic uptake of most neurotransmitters)		
	SLC6A4	Solute carrier family 6 member 4 (serotonin)		
	SNCAIP	synuclein alpha interacting protein		
	SYNGR3	Synaptogyrin 3 (synaptic vesicles)		
	TVP23B, C	*Trans*-Golgi network vesicle protein 23 homolog B and C		
	VAPA	VAMP-associated protein A		
miR-129-2-3p	GABRA1	Gamma-aminobutyric acid type A receptor alpha 1 subunit	GABRA1	Gamma-aminobutyric acid (GABA) A receptor, alpha 1
	KCNB1	Potassium voltage-gated channel subfamily B member 1	SCN3B	Sodium channel, voltage-gated, type III, beta
	MPZL1	Myelin protein zero like 1		
	NPTN	Neuroplastin (synaptic membrane Ig)		
	SACS	Sacsin molecular chaperone		
	SCN3B	Sodium voltage-gated channel beta subunit 3		
miR-130b-3p	CLCN3	Chloride voltage-gated channel 3	CLCN3	Chloride voltage-gated channel 3
	CLTC	Clathrin heavy chain	NEUROG1	Neurogenin 1
	GJA1	Gap junction protein alpha 1		
	KCNA4	Potassium voltage-gated channel subfamily A member 4		
	NEUROG1	Neurogenin 1		
	RTN1	Reticulon 1 (neuroendocrine secretion)		
	STON2	Stonin 2 (clathrin-associated)		
	SYBU	Syntabulin (axonal transport)		
miR-135a-5p	CACNA1D	Calcium voltage-gated channel subunit alpha 1 D	CACNA1D	Calcium channel, voltage-dependent, L type, alpha 1D subunit
	CACNA1E	Calcium voltage-gated channel subunit alpha 1 E	CPLX1	Complexin 1 (binding SNARE, synaptic transport)
	CPLX1	Complexin 1 (synaptic vesicles)	KCNAB3	Potassium voltage-gated channel subfamily A regulatory beta subunit 3
	CPLX2	Complexin 2 (synaptic vesicles)	KCND1	Potassium voltage-gated channel subfamily D member 1
	GRIA3	Glutamate ionotropic receptor AMPA type subunit 3	KCNJ6	Potassium voltage-gated channel subfamily J member 6
	GRID2	Glutamate ionotropic receptor delta type subunit 2	KCNS3	Potassium voltage-gated channel, modifier subfamily S, member 3
	KCNAB3	Potassium voltage-gated channel subfamily A regulatory beta subunit 3	NTNG1	Netrin G1 (axon guidance)
	KCNB1	Potassium voltage-gated channel subfamily B member 1	SLC24A2	Solute carrier family 24 member 2 (calcium/cation antiporter)
	KCNN3	Potassium calcium-activated channel subfamily N member 3	SLC5A7	Solute carrier family 5 (sodium/choline cotransporter), member 7
	KCNQ5	Potassium voltage-gated channel subfamily Q member 5	SLC6A5	Solute carrier family 6 member 5 (glycine)
	KCNS3	Potassium voltage-gated channel modifier subfamily S member 3	SYT3	Synaptotagmin 3
	NTNG1	Netrin G1 (axon guidance)	TNPO1	Transportin 1
	SEMA3A	Semaphorin 3A		
	SLC24A2	Solute carrier family 24 member 2 (calcium/cation antiporter)		
	SLC9A9	Solute carrier family 9 member A9 (sodium/proton exchanger)		
	SV2B	Synaptic vesicle glycoprotein 2B		
	SYT2	Synaptotagmin 2		
	SYT3	Synaptotagmin 3		
miR-140-5p	-		–	
miR-142-3p	CLDN12	Claudin 12 (tight junctions)	–	
	CLOCK	Clock circadian regulator		
	CLTA	Clathrin light chain A		
miR-142-5p	CBLN4	Cerebellin 4 precursor (synapse development)	NEDD1	Neural precursor cell expressed, developmentally downregulated 1
	CNTN1	Contactin 1 (neuronal adhesion)	SEPT2	Septin 2 (axon growth)
	NENF	Neudesin neurotrophic factor	SLC12A1	Solute carrier family 12 (sodium/potassium/chloride transporter), member 1
	SACS	Sacsin molecular chaperone	SLCO1B2	Solute carrier organic anion transporter family, member 1B2
	SLC12A2	Solute carrier family 12 member 2 (sodium, chloride reabsorption)		
	SLC18A2	Solute carrier family 18 member A2 (monoamines)		
	SLC24A2	Solute carrier family 24 member 2 (calcium/cation antiporter)	-	
miR-193a-5p	CLCA2	Chloride channel accessory 2	CACNB4	Calcium voltage-gated channel auxiliary subunit beta 4
	NETO2	Neuropilin and tolloid like 2 (axon guidance)		
miR-203a-3p	CAB39	Calcium binding protein 39	CACNA2D1	Calcium voltage-gated channel auxiliary subunit alpha 2 delta 1
	CLSTN3	Calsyntenin 3 (calcium-mediated postsynaptic signals)	GABARAPL1	GABA type A receptor associated protein like 1
	MYEF2	Myelin expression factor 2	GABRA1	Gamma-aminobutyric acid type A receptor alpha 1 subunit
	NOCT	Nocturnin (circadian rhythm)	SCN2A	Sodium voltage-gated channel alpha subunit 2
	OLFM3	Olfactomedin 3	SEMA5A	Semaphorin 5A (axon guidance)
	PSD3	Pleckstrin and Sec7 domain containing 3 (endocytosis)	SLC12A2	Solute carrier family 12 member 2 (Na/K/2Cl cotransporter)
	SEMA5A	Semaphorin 5A (axon guidance)	SLC9A5	Solute carrier family 9 member A5 (Na^+^/H^+^)
	SGMS2	Sphingomyelin synthase 2		
miR-484	KCNJ14	Potassium voltage-gated channel subfamily J member 14		
	TNR	Tenascin R (axon growth)		
miR-490-5p	-		-	
miR-539-5p	GABRA4	Gamma-aminobutyric acid type A receptor alpha 4 subunit	NELL2	Neural EGFL like 2
	KCNB1	Potassium voltage-gated channel subfamily B member 1	SEMA3D	Semaphorin 3D (axon guidance)
	KCNG3	Potassium voltage-gated channel modifier subfamily G member 3		
	NAV1	Neuron navigator 1		
	NELL2	Neural EGFL like 2		
	NEUROD4	Neuronal differentiation 4		
	OPRM1	Opioid receptor mu 1		
	SEMA3A	Semaphorin 3A (axon guidance)		
	SLC1A2	Solute carrier family 1 member 2 (glutamate uptake)		
	SLC2A14	Solute carrier family 2 member 14 (glucose)		
	SLC5A7	Solute carrier family 5 member 7 (choline uptake)		
	SNAP29	Synaptosome-associated protein 29		

*Brain physiology-, function-, and development-related predicted targets of miRNAs identified with altered regulation by miRNA sequencing. miRNA targets selected from a list produced by the MirTarget search tool (http://mirdb.org/cgi-bin/search.cgi) individually for humans and rats with a target score above 90 for all miRNAs with common dysregulation in mTLE/HS patients and TLE-like rats (3 months after SE).*

Similar to target prediction, humans had more affected pathways identified by *DIANA-mirPath* v3 software (Vlachos et al., 2015) compared with rat prediction data (Supplementary Table S5). In both cases, hundreds of genes undergo regulation by the combined force of miRNAs with shared dysregulation in mTLE patients and rats. Pathways common for both species predominantly comprised signaling pathways (Rap1, Ras, TGF-β, ubiquitin, etc.), however, circadian rhythm and gap junctions were also affected. In humans, this set of miRNAs also regulated the expression of genes involved in neurotransmitter synapses (dopaminergic, glutamatergic, and cholinergic) and axon guidance, while affecting the synaptic vesicle trafficking in rats.

## Discussion

The analysis of miRNA expression profile in brain tissues increasingly assists in the understanding of the biological processes involved in epileptogenesis and ictogenesis. However, previous comparative studies have explored the miRNA profiles mostly in patient samples affected by pharmacotherapies or models of epilepsy (e.g., cell culture AND animals), which do not correspond to the human biochemistry of the disease exactly. This limitation can be overcome by miRNA profile comparison of patients and model organisms that may reveal specific miRNAs playing an essential role in the onset or development of mTLE. Here, we compared the miRNA profiles of patients and rat models of epilepsy analyzed with the same workflow. This approach reduced technological bias and enhanced the accuracy of discovered miRNAs.

In our analyses of MPS results, we focused on the effect of the onset age on the differential miRNA expression, which seems to be smaller in mTLE/HS patients since 49 miRNAs show altered expression in all onset categories (Figure 2). On the contrary, the onset age in the rat model strongly determines the changes in miRNA expression related to post-SE epilepsy, and the profiling study of the animal model revealed only a limited number of dysregulated miRNAs. This difference in miRNA expression between human and model might arise from multiple sources. For instance, tissue samples originated from patients long into their condition (the period from diagnosis to surgery ranges from 1 to 45 years), which means a long and variable progression of the disease (e.g., development of comorbidities or brain damage) and exposure to multiple AEDs. Exposure to AEDs might increase the variability between humans and rats since patients received at the time of surgery on average two AEDs. Levetiracetam and carbamazepine were commonly administered to patients across all age groups. Nevertheless, the inter-individual AED variability might have introduced inconsistency in patient miRNA levels unrelated to onset age (Supplementary Tables S1, S2). We also addressed a distinct subpopulation of mTLE patients, which shares similar features—drug resistance and HS. In the rat model, potential bias increasing the influence is suppressed, and hence, the P12 and P60 groups differed solely in the age of SE induction and its consequences. We used age- and gender-matched controls for each age group, animals were not exposed to any medications besides those necessary for the model development, and they lived in a controlled environment. Furthermore, the outcome of SE induction at P12 in rats typically results in a slightly different condition than the model of SE in adult animals. Unlike adults, animals with SE at P12 develop only electrographic seizures at the chronic stage of the disease (motor seizures are not present), and they show a more discrete level of brain damage even though similar structures are affected (Nairismägi et al., 2006; Kubová and Mareš, 2013). On the other hand, induction of SE during brain development results in early manifestations of comorbidities such as impaired learning and memory problems (Rutten et al., 2002; Kubová et al., 2004; Mikulecká et al., 2019). These differences arising from the onset age in our model might explain the difference in miRNA profiles between rats with infant- and adult-onset TLEs.

Our profiling of miRNA expression in brain tissues discovered over a hundred of miRNAs with altered expression in patients with mTLE/HS (Bencurova et al., 2017). Out of these miRNAs, 10 were also dysregulated in rats with onset in adulthood. In contrast, only miR-140-5p and miR-135a-5p showed concordant dysregulation in rats with onset in infancy (Table 1 and Figure 2). Though the overlap with early-onset TLE in rats is minimal in our data, miR-140-5p shows elevated expression specific for this age group of rats and patients with adolescent-onset mTLE, which would be neglected if compared solely to the adult-onset model. The other reason for the limited overlap of miRNA profiles between the human and early-onset model of TLE might lie in the age difference – even though categorized, human patients underwent surgery with a large delay from the beginning of the disease after extensive medication. Moreover, the resemblance of human and rat with adult-onset miRNA profiles might be partially due to the presence of severe brain damage in these rats, indicating better resemblance of this model to patients with HS in mTLE/HS (Turski et al., 1986). Taken together, although our study showed cardinal differences between human and rat miRNA profiles in hippocampi, the results indicate that miRNA expression changes in mTLE/HS patients are more similar to rats with onset in adulthood than rats with onset in the infancy.

Nevertheless, almost all miRNAs detected by MPS and listed in Table 1 were previously associated with epilepsy showing the same trend of dysregulation either in both patients and animal models (miR-129-2-3p, miR-135-5p and miR-193a-5p) or in just animal models of epilepsy (miR-140-5p, miR-142-3p, miR-142-5p, miR-203a-3p, and miR-539) (Kan et al., 2012; Risbud and Porter, 2013; Gorter et al., 2014; Kretschmann et al., 2014). In the case of miR-130b-3p, dubious results were previously reported showing both upregulation and downregulation of this miRNA in both patient and animal models of TLE (Liu et al., 2010; McKiernan et al., 2012a,b; Kaalund et al., 2014). let-7b-3p was reported by Risbud and colleagues as downregulated in rats with pilocarpine-induced SE, while miR-484 and miR-490-5p have not been associated with epilepsy so far (Supplementary Table S6). Additionally, we found an opposite significant dysregulation of seven miRNAs between patients and rats (Table 2). We did not study the reason for these opposite expressions and can only speculate that they arise from a specific reaction of the rat model on TLE onset or the specificity of this animal epilepsy model. The majority of these contradicting miRNAs were previously associated with epilepsy only in animal models. Kan et al. (2012) observed (2012), similarly to our data, the elevation of miR-301a-3p in their study of mTLE patients. On the other hand, miR-187-3p and miR-211-5p were previously identified as downregulated in human patients, which corresponds with the trend of dysregulation we observed in the rat model rather than in patients (McKiernan et al., 2012a; Kaalund et al., 2014).

This cross-comparison study of our previous miRNA profiling analyses showed that the expression of miR-142-5p was significantly dysregulated in the MPS results of patients (in all age groups) and rats with seizure onset in adulthood, but it was validated only in the patient group. To identify the potential targets of this miRNA, we employed miRDB software, which predicts about 300 potential mRNA targets involved in various pathways (e.g., signaling and cell cycle). Literature indicates that these miRNA target genes were included in immunomodulatory pathways. These studies showed in a functional experiment that miR-142-5p negatively regulates the expression of pro-inflammatory agents, interleukin 6 (IL-6), or high mobility group box (HMGB) gene, which are considered pro-convulsive (Kretschmann et al., 2014; Britton, 2016). This evidence supports the idea that autoimmunity plays a significant role in ictogenesis and epileptogenesis and shall be considered in epilepsy management. Besides, ILAE has recognized autoimmune epilepsy as a distinct entity and included it into classification since 2017 (Britton, 2016).

Another miRNA with great influence on TLE might be miR-135a-5p, which was significantly altered in patients and both rat models of mTLE. However, we did not validate this miRNA by miQPCR due to its low amount in samples. Based on the literature, this miRNA mediates the expression of various proteins included in brain function and neuron development, e.g., serotonin receptor and transporter (Artigas et al., 2018) or complexin 1/2 in the amygdala (Mannironi et al., 2018). It also regulates developmental axon growth and branching, cortical neuronal migration, or regeneration of retinal ganglion cell axons (van Battum et al., 2018). Besides, recent findings revealed that miR-135a-5p affects spontaneous recurrent seizures in the TLE model (Vangoor et al., 2019), which confirms our finding.

Besides, miR-142-5p, miR-142-3p, and miR-129-2-3p have also altered expressions across all patient categories and adult-onset TLE in rats. Functional studies showed that miR-142-3p affects not only immunomodulation via control of IL-1β and IL-10 but also the expression of D1 dopaminergic receptors while suppressing neuronal growth (Tobón et al., 2012; Mandolesi et al., 2017). In the case of miR-129-2-3p, our target prediction indicated regulation of γ-aminobutyric acid receptor 1 (GABRA1) and sodium voltage-gated channel common for both humans and rats (Table 2). Previous experiments on cell cultures suggest that miR-129-2-3p represses expression of caspase 6 and spleen tyrosine kinase (SYK) (Huang et al., 2019; Umehara et al., 2019).

Among limitations of this study belongs the age discrepancy between the epilepsy onset and the resection of epileptic foci in patients, which is different from the condition in the rat model of epilepsy. Another potential drawback is patient classification based only on the age onset since different classifications (e.g., etiology based) might yield different results. Due to the small amount of unique patient samples and limited sensitivity of miQPCR, we did not validate discovered miRNAs. Finally, predicted gene targets of discussed miRNAs were generated *in silico*, and they require validation in brain tissues by functional experiments.

In summary, our cross-comparison study compared miRNA profiles of mTLE/HS patients with two different age-onset rat models of TLE. This comparison was focused on both miRNA profiles of all patients and patients categorized based on epilepsy onset age, which plays a substantial role in disorder outcome. These analyses confirmed particular dysregulation of miRNA expression in different onset ages and discovered several miRNAs, which might be truly connected to epileptogenesis and ictogenesis in both patients and animal models. Our analyses also showed that rats with TLE onset in adulthood showed greater resemblance to miRNA profiles of mTLE/HS patients than rats with onset in infancy. Finally, our study enhanced insight into the general knowledge of epilepsy and should be considered in the planning of future epilepsy experiments with animal models.

## Data Availability Statement

The datasets generated for this study can be found in the GEO database (accession numbers GSE99455 and GSE124332).

## Ethics Statement

Written informed consent was obtained from the individual(s) and/or minor(s)’ legal guardian/next of kin for the publication of any potentially identifiable images or data included in this article. The studies involving human participants were reviewed and approved by the Ethical Committee of St. Anne’s University Hospital in Brno (approval number 9G/2015-KS). The patients/participants provided their written informed consent to participate in this study. The animal study was reviewed and approved by the Ethical Committee of the Czech Academy of Sciences (approval number 128/2013).

## Author Contributions

This manuscript has been read and approved by all named authors and all authors agreed with the order of authors listed in the manuscript. The authors consent to take public responsibility for the content of the manuscript and confirm that the manuscript was prepared following ethical guidelines and regulations of their institutions concerning intellectual property. All authors have had access to all the data in the study and contributed to the study design, data acquisition, data analysis, interpretation, drafting, and revising the manuscript.

## Conflict of Interest

The authors declare that the research was conducted in the absence of any commercial or financial relationships that could be construed as a potential conflict of interest.
