# Supplementary material for: Epilepsy miRNA Profile Depends on the Age of Onset in Humans and Rats
Source: Front Neurosci. 2020 Sep 15;14:924. doi: 10.3389/fnins.2020.00924 (PMC7522367; doi:10.3389/fnins.2020.00924)
Supplement: Supplementary file 1 [file Data_Sheet_1.pdf]

Supplementary Material

1. Supplementary Tables

Table S1 – Clinical information about mTLE patients and autopsy controls – mTLE/HS – mesial temporal lobe epilepsy with hippocampal sclerosis [1]; + presence or / absence of hippocampal sclerosis (HS); AEDs – antiepileptic drugs at the time of surgery listed in parenthesis in the last column, CBZ carbamazepine, DZP diazepam, LEV levetiracetam, LTG lamotrigine, PHB phenobarbital, PHT phenytoin, TPM topiramate, ZNS zonisamide

patient/ control	age	HS	analyzed hippocampus*	diagnosis to surgery/ death to autopsy	sample storage	age of first seizure	mTLE precipitating event/ cause of death - controls	Engel score (AEDS)
mTLE /HS	40-50	+	Right	29 years	6 years	10-20	meningitis	IA (LTG)
mTLE /HS	40-50	+	Left	33 years	5,5 years	10-20	febrile seizures	IA (PHB, LEV, LTG)
mTLE /HS	20-30	+	Left	22 years	4,5 years	0-10	-	IC (CBZ, LTG)
mTLE /HS	50-60	+	Left	28 years	3,5 years	20+	meningitis	IA (CBZ, ZNS)
mTLE /HS	40-50	+	Left	45 years	3 years	0-10	-	IA (LTG, CBZ, LEV, DZP)
mTLE /HS	30-40	+	Left	21 years	3,5 years	10-20	febrile seizures	IC (TPM)
mTLE /HS	40-50	+	Left	30 years	3 years	10-20	-	IA (LEV, LTG)
mTLE /HS	40-50	+	Left	21 years	3 years	10-20	meningoencephalitis	IIIA (CBZ)
mTLE /HS	40-50	+	Left	43 years	3,5 years	0-10	-	IC (CBZ, LTG)
mTLE /HS	40-50	+	Right	1 year	3 years	20+	-	IA (LEV, CBZ)
mTLE /HS	30-40	+	Right	22 years	2 years	10-20	febrile seizures	IA (LTG, LEV)
mTLE /HS	30-40	+	Right	26 years	2,5 years	0-10	-	IC (PHT, ZNS, LEV)
mTLE /HS	40-50	+	Left	10 years	2 years	20+	meningitis	IA (ZNS, LEV)
mTLE /HS	30-40	+	Left	30 years	2 years	0-10	meningoencephalitis	IA (TPM, LEV)
mTLE /HS	20-30	+	Left	5 years	2 years	20+	febrile seizures	IA (LEV, CBZ)
mTLE /HS	30-40	+	Left	5 years	2 years	20+	commotio cerebri	IA (CBZ, LEV)
Ctrl	60-70	/	Right	12 hours	<1 year	-	bronchopnemonia	
Ctrl	60-70	/	Right	14 hours	<1 year	-	heart failure	
Ctrl	50-60	/	Left	28 hours	<1 year	-	myocardial infarction	
Ctrl	70-80	/	Right	4 hours	<1 year	-	heart failure	
Ctrl	70-80	/	Left	4 hours	<1 year	-	heart failure	
Ctrl	50-60	/	Left	6 hours	<1 year	-	heart failure	
Ctrl	30-40	/	Left	14 hours	<1 year	-	heart failure	
Ctrl	40-50	/	Right	11 hours	<1 year	-	heart failure	

* epilepsy foci - patients

Table S2

Overview of antiepileptic medication. Number of patients in individual onset age categories (childhood, adolescent, adult) taking antiepileptic drug (AED) at the time of surgery (on average 2 AEDs per patient) and throught the epilepsy duration (on average 5 AEDs per patient)

	AEDs at the time of surgery				*AEDs taken since the epilepsy onset*			
**Antiepileptic drug (AED)**	**Childhood (0-10 years)**	**Adolescent (10-20 years)**	**Adult (20+ years)**	**% of all AEDs**	**Childhood (0-10 years)**	**Adolescent (10-20 years)**	**Adult (20+ years)**	**% of all AEDs**
**carbamazepine**	3	1	4	24	5	3	5	15
**clonazepam**	0	0	0	0	2	0	1	3
**diazepam**	1	0	0	3	2	1	0	3
**gabapentin**	0	0	0	0	0	1	0	1
**lacosamide**	0	0	0	0	0	0	1	1
**lamotrigine**	3	4	0	21	4	6	2	14
**levetiracetam**	3	3	4	30	4	4	5	15
**oxcarbazepine**	0	0	0	0	1	0	0	1
**phenobarbital**	0	1	0	3	0	3	0	3
**phenytoin**	1	0	0	3	3	2	1	7
**pregabaline**	0	0	0	0	0	1	1	2
**primidone**	0	0	0	0	1	2	2	6
**retigabine**	0	0	0	0	0	1	0	1
**sulthiam**	0	0	0	0	0	1	0	1
**topiramate**	1	1	0	6	2	3	1	7
**valproic acid**	0	0	0	0	3	2	4	10
**vigabatrin**	0	0	0	0	0	0	1	1
**zonisamide**	1	0	2	9	2	0	3	6

Table S3 – miRNAs significantly dysregulated in mTLE/HS patients - miRNAs identified as significantly altered in mTLE/ HS patients compered with controls by miRNA sequencing (miR-Seq). The list contains all miRNAs that reached fold-change above 1.4, p-value <0.05 and threshold of 500 reads in all human hippocampal samples [1]. ** p-value< 0.01; * 0.01<p-value<0.05; - p-value> 0.05; FC fold change

Childhood onset (1st seizure < 10)			Adolescence onset (1st seizure 11-19)			Adult onset (first seizure > 20)		
**miRNA**	**p val**	**FC**	**miRNA**	**p val**	**FC**	**miRNA**	**p val**	**FC**
hsa-miR-100-5p	**	1,50	hsa-miR-100-5p	**	1,75	hsa-miR-100-5p	*	1,69
hsa-miR-103a-3p	**	-1,47	hsa-miR-103a-3p	**	-1,48	hsa-miR-1250-5p	*	-1,77
hsa-miR-124-3p	**	1,46	hsa-miR-1180-3p	*	-1,57	hsa-miR-1260a	**	2,19
hsa-miR-1249-3p	**	-2,54	hsa-miR-124-3p	**	1,59	hsa-miR-1260b	**	1,96
hsa-miR-1250-5p	*	-1,58	hsa-miR-1249-3p	**	-2,72	hsa-miR-1275	**	5,82
hsa-miR-1260a	**	2,27	hsa-miR-1260a	**	2,40	hsa-miR-129-2-3p	**	3,10
hsa-miR-1260b	**	2,71	hsa-miR-1260b	**	2,89	hsa-miR-129-5p	*	1,61
hsa-miR-126-3p	**	-1,52	hsa-miR-126-3p	**	-1,51	hsa-miR-130b-3p	**	-1,45
hsa-miR-1275	**	5,58	hsa-miR-1275	**	5,93	hsa-miR-132-5p	*	-1,48
hsa-miR-129-2-3p	**	4,11	hsa-miR-128-1-5p	*	-1,50	hsa-miR-135a-5p	*	1,75
hsa-miR-1298-3p	*	-1,45	hsa-miR-129-2-3p	**	4,12	hsa-miR-138-2-3p	**	-1,68
hsa-miR-1298-5p	**	-2,68	hsa-miR-1298-5p	**	-1,98	hsa-miR-142-3p	**	2,19
hsa-miR-1306-5p	*	-1,56	hsa-miR-1306-5p	**	-2,11	hsa-miR-142-5p	**	1,90
hsa-miR-1307-5p	**	1,62	hsa-miR-1307-5p	**	1,94	hsa-miR-144-3p	*	2,34
hsa-miR-132-5p	**	-1,71	hsa-miR-132-3p	**	-1,63	hsa-miR-144-5p	*	4,35
hsa-miR-134-5p	**	-1,46	hsa-miR-132-5p	**	-1,68	hsa-miR-1468-5p	**	-1,59
hsa-miR-138-2-3p	**	-2,21	hsa-miR-134-5p	**	-1,55	hsa-miR-150-5p	**	1,87
hsa-miR-142-3p	**	2,09	hsa-miR-136-3p	*	1,54	hsa-miR-152-3p	*	1,54
hsa-miR-142-5p	**	2,10	hsa-miR-138-2-3p	**	-2,47	hsa-miR-153-3p	*	1,71
hsa-miR-144-3p	**	4,31	hsa-miR-139-3p	**	-1,42	hsa-miR-181a-5p	**	-2,16
hsa-miR-144-5p	**	6,11	hsa-miR-140-5p	*	1,41	hsa-miR-181b-5p	**	-1,92
hsa-miR-1468-5p	**	-2,58	hsa-miR-142-3p	**	2,42	hsa-miR-182-5p	**	2,12
hsa-miR-148b-3p	*	1,47	hsa-miR-142-5p	**	1,87	hsa-miR-184	**	-8,49
hsa-miR-149-5p	**	1,69	hsa-miR-144-3p	**	3,86	hsa-miR-1911-5p	**	-12,00
hsa-miR-150-5p	**	1,53	hsa-miR-144-5p	**	5,84	hsa-miR-195-5p	**	1,82
hsa-miR-151b	**	-1,71	hsa-miR-1468-5p	**	-3,82	hsa-miR-19a-3p	**	2,38
hsa-miR-181a-3p	*	1,59	hsa-miR-148b-3p	*	1,50	hsa-miR-210-3p	*	-1,86
hsa-miR-181a-5p	**	-1,71	hsa-miR-149-5p	**	1,67	hsa-miR-211-5p	**	-4,96
hsa-miR-181b-5p	**	-1,50	hsa-miR-150-5p	**	1,44	hsa-miR-214-3p	*	1,81
hsa-miR-182-5p	**	1,75	hsa-miR-151b	**	-1,67	hsa-miR-218-5p	*	-1,45

hsa-miR-184	**	-5,59	hsa-miR-181a-3p	**	1,85	hsa-miR-219a-2-3p	**	-2,81
hsa-miR-1911-5p	**	-24,00	hsa-miR-181a-5p	*	-1,77	hsa-miR-221-3p	**	-2,10
hsa-miR-193a-5p	**	1,84	hsa-miR-184	**	-6,02	hsa-miR-23a-3p	**	1,51
hsa-miR-193b-3p	**	1,92	hsa-miR-187-3p	*	1,73	hsa-miR-29b-2-5p	**	-1,51
hsa-miR-195-5p	**	1,80	hsa-miR-1911-5p	**	-12,00	hsa-miR-301a-3p	**	2,26
hsa-miR-199b-3p	**	-1,53	hsa-miR-193a-5p	*	1,83	hsa-miR-320e	**	-5,64
hsa-miR-19a-3p	**	1,97	hsa-miR-193b-3p	**	1,88	hsa-miR-330-5p	**	-1,52
hsa-miR-19b-3p	**	1,86	hsa-miR-195-5p	**	1,82	hsa-miR-339-5p	**	1,76
hsa-miR-203a-3p	**	1,99	hsa-miR-199b-3p	**	-1,51	hsa-miR-33a-5p	*	2,54
hsa-miR-210-3p	**	-1,67	hsa-miR-19a-3p	**	2,63	hsa-miR-342-3p	**	1,83
hsa-miR-214-3p	**	1,55	hsa-miR-19b-3p	**	2,46	hsa-miR-34b-5p	**	-3,23
hsa-miR-218-5p	**	-1,93	hsa-miR-203a-3p	**	1,97	hsa-miR-34c-5p	*	-2,10
hsa-miR-219a-2-3p	*	-1,80	hsa-miR-210-3p	**	-1,59	hsa-miR-3653-3p	**	1,75
hsa-miR-219a-5p	**	1,93	hsa-miR-211-5p	**	-3,29	hsa-miR-365b-3p	**	1,88
hsa-miR-221-3p	**	-1,97	hsa-miR-212-3p	**	-2,22	hsa-miR-374b-5p	**	2,73
hsa-miR-223-3p	**	2,01	hsa-miR-214-3p	*	1,68	hsa-miR-375	**	-1,60
hsa-miR-22-3p	**	-1,81	hsa-miR-218-5p	**	-1,78	hsa-miR-376a-3p	**	1,97
hsa-miR-23a-3p	**	1,70	hsa-miR-219a-5p	**	2,65	hsa-miR-424-5p	**	2,02
hsa-miR-23b-3p	**	1,46	hsa-miR-221-3p	**	-2,06	hsa-miR-4286	**	1,96
hsa-miR-24-1-5p	*	-1,52	hsa-miR-222-3p	**	-1,42	hsa-miR-4301	**	-2,44
hsa-miR-27a-3p	**	1,67	hsa-miR-223-3p	**	2,08	hsa-miR-4443	**	3,54
hsa-miR-296-5p	**	1,44	hsa-miR-22-3p	**	-1,83	hsa-miR-4454	**	2,58
hsa-miR-29a-3p	**	-1,40	hsa-miR-23a-3p	**	1,57	hsa-miR-4459	*	-1,62
hsa-miR-29b-1-5p	**	-2,19	hsa-miR-23b-3p	**	1,52	hsa-miR-451a	**	3,46
hsa-miR-29b-2-5p	**	-1,95	hsa-miR-27a-3p	**	1,89	hsa-miR-484	**	1,46
hsa-miR-301a-3p	**	1,79	hsa-miR-296-5p	**	1,56	hsa-miR-487a-3p	**	2,51
hsa-miR-30b-5p	**	1,50	hsa-miR-29a-3p	**	-1,42	hsa-miR-490-3p	**	2,60
hsa-miR-30e-3p	**	1,80	hsa-miR-29b-1-5p	**	-1,98	hsa-miR-490-5p	**	1,44
hsa-miR-3200-3p	**	1,48	hsa-miR-29b-2-5p	**	-1,98	hsa-miR-495-3p	*	1,45
hsa-miR-320e	**	-4,39	hsa-miR-301a-3p	**	1,91	hsa-miR-5010-3p	*	1,41
hsa-miR-326	**	1,42	hsa-miR-30b-5p	**	1,61	hsa-miR-5100	**	1,99
hsa-miR-328-3p	**	-1,74	hsa-miR-30e-3p	**	1,80	hsa-miR-532-3p	*	1,53
hsa-miR-339-5p	**	2,22	hsa-miR-3200-3p	**	1,75	hsa-miR-567	**	1,74
hsa-miR-33a-5p	**	2,63	hsa-miR-320b	**	-1,46	hsa-miR-598-3p	**	-1,46
hsa-miR-342-3p	**	1,95	hsa-miR-320c	*	-1,57	hsa-miR-607	*	-1,64
hsa-miR-34a-5p	**	-1,57	hsa-miR-320e	**	-4,39	hsa-miR-6087	*	2,21
hsa-miR-34b-3p	*	-2,93	hsa-miR-323a-3p	*	-1,57	hsa-miR-6131	**	-9,38
hsa-miR-34b-5p	**	-6,93	hsa-miR-328-3p	**	-2,16	hsa-miR-663a	*	-4,13
hsa-miR-34c-5p	**	-4,45	hsa-miR-339-5p	**	2,17	hsa-miR-664a-3p	**	1,42

hsa-miR-3605-3p	**	-3,26	hsa-miR-33a-5p	**	3,17	hsa-miR-668-3p	*	1,99
hsa-miR-3613-5p	**	-1,55	hsa-miR-342-3p	**	1,94	hsa-miR-6716-3p	*	-1,95
hsa-miR-3653-3p	**	1,59	hsa-miR-34a-5p	**	-1,61	hsa-miR-7110-3p	**	-3,37
hsa-miR-365b-3p	**	2,22	hsa-miR-34b-3p	*	-2,56	hsa-miR-759	**	4,15
hsa-miR-374b-5p	**	3,33	hsa-miR-34b-5p	*	-6,93	hsa-miR-7-5p	*	-1,52
hsa-miR-375	*	-2,27	hsa-miR-34c-5p	**	-3,02	hsa-miR-766-3p	*	1,54
hsa-miR-382-5p	**	-1,73	hsa-miR-3605-3p	**	-3,65	hsa-miR-874-3p	**	1,83
hsa-miR-409-3p	**	-1,42	hsa-miR-3648	**	-1,87	hsa-miR-92b-3p	**	1,97
hsa-miR-424-3p	**	1,70	hsa-miR-3653-3p	**	1,59	hsa-miR-92b-5p	*	2,00
hsa-miR-424-5p	**	2,10	hsa-miR-365b-3p	**	2,22	hsa-miR-99a-5p	**	2,34
hsa-miR-4286	**	2,74	hsa-miR-374a-3p	**	1,49	hsa-miR-99b-3p	**	-1,59
hsa-miR-4301	**	-2,87	hsa-miR-374a-5p	*	1,42			
hsa-miR-432-5p	**	-1,66	hsa-miR-374b-5p	**	3,33			
hsa-miR-433-3p	**	-1,69	hsa-miR-375	**	-2,48			
hsa-miR-4443	**	5,00	hsa-miR-382-5p	**	-1,76			
hsa-miR-4454	**	5,42	hsa-miR-409-3p	**	-1,62			
hsa-miR-4508	**	3,55	hsa-miR-424-5p	**	2,69			
hsa-miR-451a	**	5,21	hsa-miR-4286	**	2,72			
hsa-miR-485-3p	**	-1,94	hsa-miR-4301	**	-3,81			
hsa-miR-485-5p	*	-1,57	hsa-miR-432-5p	**	-1,88			
hsa-miR-487a-5p	**	-1,60	hsa-miR-433-3p	**	-1,95			
hsa-miR-490-3p	**	1,82	hsa-miR-4443	**	4,23			
hsa-miR-493-5p	*	-2,04	hsa-miR-4454	**	5,74			
hsa-miR-500a-3p	**	1,73	hsa-miR-4508	*	2,68			
hsa-miR-501-5p	**	-1,44	hsa-miR-451a	**	5,11			
hsa-miR-504-5p	*	-1,68	hsa-miR-4787-3p	*	1,64			
hsa-miR-5100	**	2,13	hsa-miR-4791	*	1,50			
hsa-miR-539-5p	**	-1,73	hsa-miR-484	**	1,40			
hsa-miR-543	*	-1,44	hsa-miR-485-3p	**	-2,63			
hsa-miR-551b-3p	**	2,17	hsa-miR-487a-3p	*	1,56			
hsa-miR-592	**	-1,43	hsa-miR-487a-5p	**	-1,86			
hsa-miR-6087	**	4,13	hsa-miR-490-3p	*	1,75			
hsa-miR-6131	**	-4,91	hsa-miR-493-5p	**	-2,61			
hsa-miR-629-5p	**	-1,81	hsa-miR-500a-3p	**	1,94			
hsa-miR-654-5p	**	-1,95	hsa-miR-501-5p	**	-2,89			
hsa-miR-655-3p	*	1,40	hsa-miR-504-5p	**	-1,77			
hsa-miR-656-3p	**	1,51	hsa-miR-5100	**	2,14			
hsa-miR-663a	**	-6,60	hsa-miR-539-5p	**	-1,86			
hsa-miR-664a-3p	**	1,50	hsa-miR-543	**	-1,96			
hsa-miR-664a-5p	**	-1,46	hsa-miR-551b-3p	**	2,48			
hsa-miR-6716-3p	**	-2,11	hsa-miR-592	**	-1,40			

hsa-miR-708-5p	*	1,41	hsa-miR-6087	**	5,58
hsa-miR-7110-3p	**	-2,82	hsa-miR-6131	**	-5,50
hsa-miR-759	**	5,46	hsa-miR-629-5p	**	-2,29
hsa-miR-7-5p	**	-1,84	hsa-miR-654-5p	**	-2,03
hsa-miR-7641	*	1,72	hsa-miR-656-3p	**	1,59
hsa-miR-766-3p	**	1,83	hsa-miR-663a	**	-6,19
hsa-miR-874-3p	**	2,49	hsa-miR-664a-3p	*	1,46
hsa-miR-887-3p	**	1,40	hsa-miR-664a-5p	**	-1,58
hsa-miR-935	*	-1,43	hsa-miR-6716-3p	**	-1,86
hsa-miR-95-3p	**	-1,87	hsa-miR-7110-3p	**	-3,26
hsa-miR-99a-3p	*	1,47	hsa-miR-759	**	5,54
hsa-miR-99a-5p	**	2,42	hsa-miR-7-5p	**	-2,14
hsa-miR-99b-3p	**	-1,55	hsa-miR-7641	*	1,83
			hsa-miR-766-3p	**	1,82
			hsa-miR-874-3p	**	2,67
			hsa-miR-887-3p	**	1,65
			hsa-miR-935	**	-1,70
			hsa-miR-95-3p	**	-2,12
			hsa-miR-99a-3p	**	1,52
			hsa-miR-99a-5p	**	2,55
			hsa-miR-99b-3p	*	-1,51

Table S4 – Regression analysis of miRNA read counts and duration of epilepsy - the relationship between read count of selected miRNAs and duration of epilepsy of patients was computed using generalized linear modelling with negative binomial assumption. The `Slope` measure the relation between read counts and dureation of epilepsy, `Standard error` shows the accuracy of the analysis, `Z-score` shows different of data points from the mean, and *p-value*.

miRNA	Slope	Stadard error	Z-score	p-value
hsa-miR-142-3p	-0.04	0.014	-3.123	0.002
hsa-miR-135a-5p	-0.03	0.011	-2.633	0.008
hsa-miR-484	0.01	0.005	2.205	0.027
hsa-miR-490-5p	-0.01	0.006	-1.959	0.050
hsa-miR-193a-5p	0.01	0.009	1.486	0.137
hsa-miR-142-5p	-0.01	0.014	-1.041	0.298
hsa-miR-203a-3p	0.01	0.008	0.682	0.495
hsa-miR-140-5p	0.00	0.007	-0.648	0.517
hsa-miR-539-5p	0.00	0.009	-0.461	0.645
hsa-miR-130b-3p	0.00	0.005	0.322	0.747
hsa-let-7b-3p	0.00	0.015	0.307	0.759
hsa-miR-129-2-3p	0.00	0.007	-0.139	0.889

Table S5 – miRNAs significantly dysregulated in post SE rats - miRNAs identified as significantly altered in rats with TLE-like syndrome compared with controls. The list contains all miRNAs that reached fold-change above 1.4, p-value <0.05 and threshold of 500 reads in all rat hippocampal samples (submitted manuscript). ** p-value< 0.01; * 0.01<p-value<0.05; - p-value> 0.05; FC fold change

**Infant (SE at P12)**			**Adult-onset (SE at P60)**		
**miRNA**	**p-value**	**FC**	**miRNA**	**p-value**	**FC**
rno-miR-135a-5p	**	1,46	let-7b-3p	**	1,58
rno-miR-140-5p	*	1,42	rno-miR-1193-3p	*	-1,51
rno-miR-148b-5p	**	2,24	rno-miR-129-2-3p	**	1,66
rno-miR-187-5p	*	-3,06	rno-miR-130b-3p	**	-3,48
rno-miR-204-5p	*	-1,66	rno-miR-133b-3p	*	-2,79
rno-miR-22-3p	*	1,89	rno-miR-135a-5p	**	1,58
rno-miR-24-2-5p	**	1,64	rno-miR-138-5p	**	-1,43
rno-miR-24-3p	*	1,50	rno-miR-142-3p	*	1,54
rno-miR-301a-3p	*	-1,46	rno-miR-142-5p	*	1,58
rno-miR-370-3p	*	1,63	rno-miR-146a-5p	**	2,14
rno-miR-431	**	2,18	rno-miR-155-5p	**	2,93
rno-miR-666-3p	*	1,67	rno-miR-17-5p	**	1,50
rno-miR-7a-1-3p	*	-1,41	rno-miR-187-3p	**	-2,05
			rno-miR-193a-5p	*	1,58
			rno-miR-203a-3p	**	1,57
			rno-miR-206-3p	*	-2,06
			rno-miR-20a-5p	*	1,42
			rno-miR-212-3p	**	1,69
			rno-miR-212-5p	**	1,47
			rno-miR-218a-5p	**	-1,52
			rno-miR-24-2-5p	*	1,50
			rno-miR-291b	*	8,91
			rno-miR-293-5p	**	7,27
			rno-miR-3120	*	3,43
			rno-miR-339-3p	*	1,45
			rno-miR-350	**	1,45
			rno-miR-3547	*	-3,36
			rno-miR-3594-5p	*	-2,09
			rno-miR-365-3p	**	1,57
			rno-miR-381-5p	**	-1,69
			rno-miR-449a-5p	*	-1,55
			rno-miR-483-5p	**	-7,29
			rno-miR-484	**	1,87
			rno-miR-490-5p	*	1,99
			rno-miR-496-5p	*	-3,77
			rno-miR-504	*	-1,53
			rno-miR-539-5p	*	-1,43
			rno-miR-676	*	-1,48
			rno-miR-764-3p	**	-2,62
			rno-miR-7a-5p	**	-1,71
			rno-miR-7b	**	-1,91

Table S6 – Pathways regulated by differentially expressed miRNAs common for mTLE/HS patients and post SE rats – DIANA-mirPath v3 software indicated putative affected pathways based on the list of all miRNAs with common dysregulation in patients and rat model. Pathways regulated by the same group of miRNAs are highlighted by shading and displayed separately for individual species (human and rat). Listed p-value describes the probability of association between a given pathway and set of miRNAs

species	KEGG pathway	p-value	# of affected genes	miRNAs involved
human	cAMP signaling pathway	0,002	69	hsa-let-7b-3p, miR-129-2-3p,-130b-3p, -135a-5p, -140-5p, -142-3p, -142-5p, -193a-5p, -203a-3p, -484, -490-5p, -539-5p
	cGMP-PKG signaling pathway	0,000	63	
	Dopaminergic synapse	0,016	47	
	Glutamatergic synapse	0,002	40	
	Cholinergic synapse	0,010	42	
	Long-term potentiation	0,006	28	
	Oxytocin signaling pathway	0,001	58	
	Regulation of actin cytoskeleton	0,002	76	
	Adherens junction	0,000	34	hsa-let-7b-3p, miR-129-2-3p,-130b-3p, -135a-5p, -140-5p, -142-3p, -142-5p, -193a-5p, -203a-3p, -484, -539-5p
	AMPK signaling pathway	0,026	43	
	Circadian rhythm	0,005	17	
	Phosphatidylinositol signaling system	0,000	31	
	Rap1 signaling pathway	0,000	74	
	Ras signaling pathway	0,001	73	
	Signaling pathways regulating pluripotency of stem cells	0,000	63	
	Sphingolipid signaling pathway	0,019	37	
	Thyroid hormone signaling pathway	0,000	46	
	Ubiquitin mediated proteolysis	0,001	49	
	Wnt signaling pathway	0,002	48	
	Amphetamine addiction	0,000	26	hsa-let-7b-3p, miR-129-2-3p,-130b-3p, -135a-5p, -140-5p, -142-5p, -193a-5p, -203a-3p, -484, -490-5p, -539-5p
	ErbB signaling pathway	0,001	35	hsa-let-7b-3p, miR-129-2-3p,-130b-3p, -135a-5p, -140-5p, -142-3p, -142-5p, -203a-3p, -484, -490-5p, -539-5p
	FoxO signaling pathway	0,001	51	hsa-let-7b-3p, miR-129-2-3p,-130b-3p, -135a-5p, -142-3p, -142-5p, -193a-5p, -203a-3p, -484, -490-5p, -539-5p
	Axon guidance	0,000	46	hsa-let-7b-3p, miR-129-2-3p,-130b-3p, -135a-5p, -140-5p, -142-3p, -142-5p, -203a-3p, -484, -539-5p
	Hedgehog signaling pathway	0,040	20	
	Hippo signaling pathway	0,001	54	
	N-Glycan biosynthesis	0,045	15	hsa-let-7b-3p, miR-129-2-3p, -135a-5p, -140-5p, -142-3p, -142-5p, -193a-5p, -203a-3p, -484, -539-5p
	Gap junction	0,019	29	
	Platelet activation	0,020	42	

	TGF-beta signaling pathway	0,001	33	hsa-let-7b-3p,miR-130b-3p, -135a-5p, -140-5p, -142-3p, -142-5p, -193a-5p, -203a-3p, -484, -539-5p
	mTOR signaling pathway	0,009	26	hsa-let-7b-3p, miR-130b-3p, -135a-5p, -142-3p, -142-5p, -203a-3p, -484, -539-5p
rat	Rap1 signaling pathway<br>Ras signaling pathway	0,019<br>0,004	22<br>26	rno-let-7b-3p, miR-129-2-3p,-130b-3p, -135a-5p, -140-5p, -142-3p, -142-5p, -193a-5p, -203a-3p, -484, -490-5p, -539-5p
	Signaling pathways regulating pluripotency of stem cells	0,002	20	rno-let-7b-3p, miR-129-2-3p,-130b-3p, -135a-5p, -142-5p, -193a-5p, -203a-3p, -484, -490-5p, -539-5p
	Synaptic vesicle cycle	0,000	14	rno-let-7b-3p, miR-129-2-3p,-130b-3p, -135a-5p, -140-5p, -142-3p, -142-5p, -203a-3p, -484, -539-5p
	FoxO signaling pathway	0,024	17	rno-let-7b-3p, miR-129-2-3p,-130b-3p, -135a-5p, -140-5p, -142-3p, -142-5p, -203a-3p, -484
	TGF-beta signaling pathway<br>Ubiquitin mediated proteolysis	0,002<br>0,000	17<br>25	rno-let-7b-3p, miR-129-2-3p,-130b-3p, -135a-5p, -140-5p, -142-3p, -142-5p, -203a-3p, -539-5p
	Circadian rhythm	0,022	7	rno-let-7b-3p, miR-130b-3p, -135a-5p, -142-5p, -193a-5p, -203a-3p
	Phosphatidylinositol signaling system	0,038	10	rno-miR-129-2-3p,-130b-3p, -135a-5p, -142-3p, -142-5p, -203a-3p, -484
	N-Glycan biosynthesis	0,014	7	rno-let-7b-3p, miR-129-2-3p,-130b-3p, -135a-5p, -193a-5p, -484
	SNARE interactions in vesicular transport	0,002	7	rno-miR-135a-5p, -140-5p, -142-3p, -142-5p, -203a-3p, -484
	Gap junction	0,028	8	rno-miR-129-2-3p,-130b-3p, -135a-5p, -140-5p, -484
	Mucin type O-Glycan biosynthesis	0,028	4	rno-miR-130b-3p, -140-5p, -142-5p

Table S7 – Literature associating identified miRNAs with epilepsy – overview of the miRNAs with common dysregulation identified in mTLE/HS patients and post-SE rats and their occurrence in the literature addressing miRNA involvement in epilepsy. -1 downregulated in epilepsy; 1 upregulated in epilepsy

**Organism**	**Rat**		**Mouse**		**Human**	
**miRNA**	expression	reference	expression	reference	expression	reference
let-7b-3p	-1	2				
miR-129-2-3p	1	3	1	4	1	1, 5
miR-130b-3p			1,-1	6, 7	1,-1	8, 9
miR-135a-5p	1	3	1	4, 10	1	5
miR-140-5p	1	3	1	4, 8		
miR-142-3p	1	2	1	4	1	1
miR-142-5p	1	2	1	4	1	1
miR-193a-5p	1	2			1	5
miR-203a-3p	1	11	1	12		
miR-484						
miR-490-5p						
miR-539-5p	-1	2				

2. Supplementary Figures

Figure S1 – motor seizure frequency in animals with adult-onset TLE

The figure shows the number of motor seizures detected in individual animals over 7 day period of video-monitoring prior to brain tissue collection 3 months after induction of status epilepticus. Animals (n = 13) were allocated numbers 2 to 14 based on the camera channels used for their monitoring. One animal was euthanized due to the tumor and one animal did not have motor any seizures during the monitoring (N10 and N6, not displayed). Animal N12 had repeated generalized tonic-clonic seizures. Animals N6,10 and 12 were excluded from further analysis (submitted manuscript).

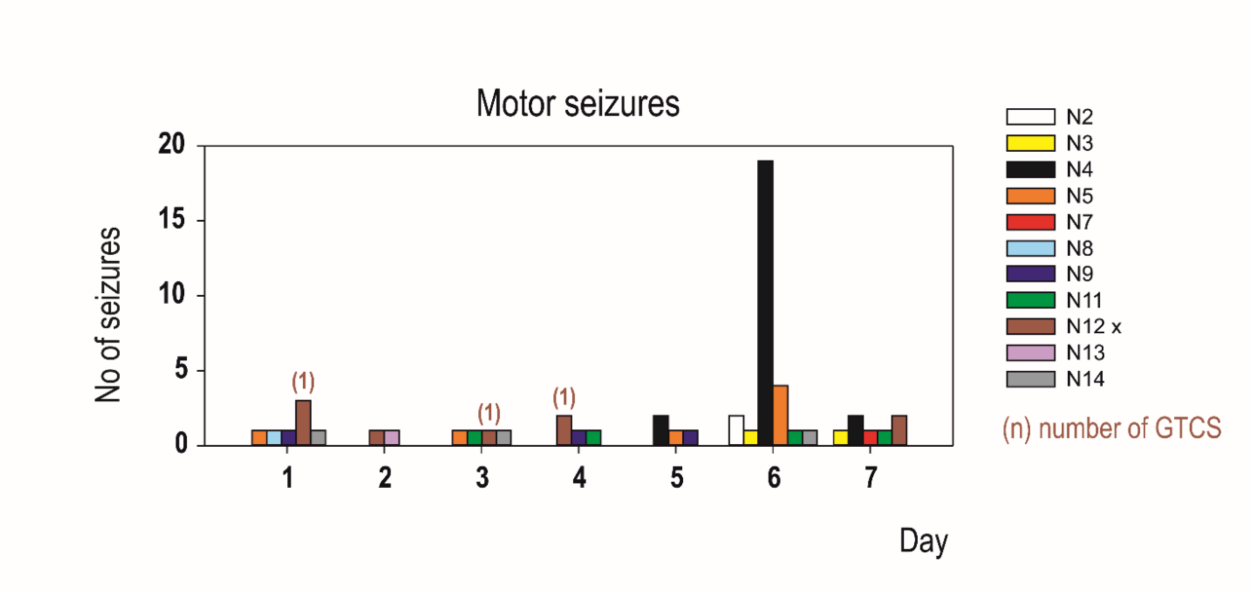

Figure S2 – Effect of epilepsy duration on miRNA expression

Figures display the miRNA read-count distribution in mTLE/HS patients based on their epilepsy duration from the first seizure until surgical resection of the epileptic foci. miRNAs identified as commonly dysregulated in patients and post-SE rats by MPS show neglected dependence on the epilepsy duration.

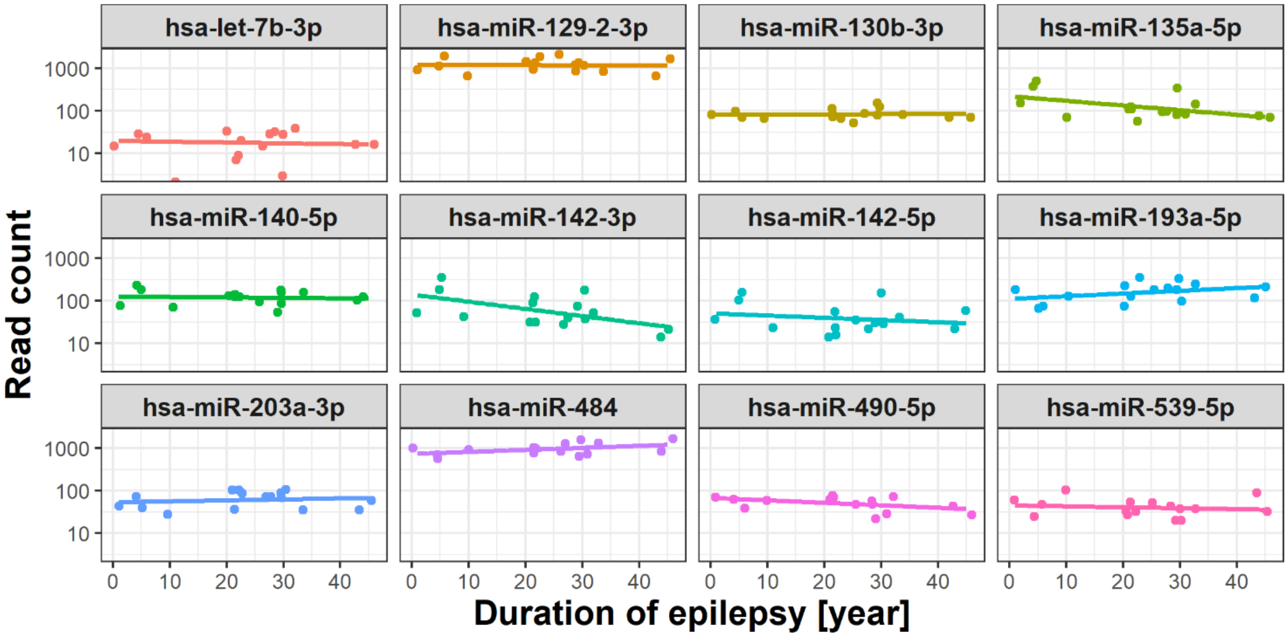

3. References

1 Bencurova P, Baloun J, Musilova K, et al (2017) MicroRNA and mesial temporal lobe epilepsy with hippocampal sclerosis: Whole miRNome profiling of human hippocampus. *Epilepsia* 58:1782–1793. https://doi.org/10.1111/epi.13870

2 Risbud RM, Porter BE (2013) Changes in MicroRNA Expression in the Whole Hippocampus and Hippocampal Synaptoneurosome Fraction following Pilocarpine Induced Status Epilepticus. *PLoS One* 8:e53464. https://doi.org/10.1371/journal.pone.0053464

3 Gorter JA, Iyer A, White I, et al (2014) Hippocampal subregion-specific microRNA expression during epileptogenesis in experimental temporal lobe epilepsy. *Neurobiol Dis* 62:508–520. https://doi.org/10.1016/j.nbd.2013.10.026

4 Kretschmann A, Danis B, Andonovic L, et al (2014) Different MicroRNA Profiles in Chronic Epilepsy Versus Acute Seizure Mouse Models. *J Mol Neurosci* 55:1–14. https://doi.org/10.1007/s12031-014-0368-6

5 Kan AA, van Erp S, Derijck AAHA, et al (2012) Genome-wide microRNA profiling of human temporal lobe epilepsy identifies modulators of the immune response. *Cell Mol Life Sci* 69:3127–3145. https://doi.org/10.1007/s00018-012-0992-7

6 Liu D-Z, Tian Y, Ander BP, et al (2010) Brain and blood microRNA expression profiling of ischemic stroke, intracerebral hemorrhage, and kainate seizures. *J Cereb Blood Flow Metab* 30:92–101. https://doi.org/10.1038/jcbfm.2009.186

7 McKiernan RC, Jimenez-Mateos EM, Sano T, et al (2012) Expression profiling the microRNA response to epileptic preconditioning identifies miR184 as a modulator of seizure-induced neuronal death. *Exp Neurol* 237:346–354. https://doi.org/10.1016/j.expneurol.2012.06.029

8 McKiernan RC, Jimenez-Mateos EM, Bray I, et al (2012) Reduced mature microRNA levels in association with dicer loss in human temporal lobe epilepsy with hippocampal sclerosis. *PLoS One* 7:e35921. https://doi.org/10.1371/journal.pone.0035921

9 Kaalund SS, Venø MT, Bak M, et al (2014) Aberrant expression of miR-218 and miR-204 in human mesial temporal lobe epilepsy and hippocampal sclerosis-Convergence on axonal guidance. *Epilepsia* 55:2017–2027. https://doi.org/10.1111/epi.12839

10 Vangoor VR, Reschke CR, Senthilkumar K, et al (2019) Antagonizing increased miR-135a levels at the chronic stage of experimental TLE reduces spontaneous recurrent seizures. *J Neurosci* 39:5064–5079. https://doi.org/10.1523/JNEUROSCI.3014-18.2019

11 Hu K, Xie Y-Y, Zhang C, et al (2012) MicroRNA expression profile of the hippocampus in a rat model of temporal lobe epilepsy and miR-34a-targeted neuroprotection against hippocampal neurone cell apoptosis post-status epilepticus. *BMC Neurosci* 13:115. https://doi.org/10.1186/1471-2202-13-115

12 Schouten M, Bielefeld P, Fratantoni SA, et al (2016) Multi-omics profile of the mouse dentate gyrus after kainic acid-induced status epilepticus. *Sci Data* 3:.

https://doi.org/10.1038/sdata.2016.68
